# Integrative UHPLC-HRMS and computational biology reveal ferroptosis and anoikis targeting by Wenpitongluo decoction in cardiorenal syndrome

**DOI:** 10.3389/fchem.2025.1617676

**Published:** 2025-07-21

**Authors:** Xinxin Mao, Shuqing Shi, Chunmei Chen, Yumeng Li, Bingxuan Zhang, Qingqiao Song

**Affiliations:** Department of Internal Medicine, Guang’anmen Hospital, China Academy of Chinese Medical Sciences, Beijing, China

**Keywords:** cardiorenal syndrome, ferroptosis, anoikis, Wenpitongluo decoction, oxidative stress, computational biology

## Abstract

**Background:**

The Wenpitongluo Decoction (WPTLD) was a classical herbal formula composed of medicinal herbs with both edible and therapeutic properties. It demonstrated clinical efficacy in treating Cardiorenal Syndrome (CRS), though its mechanism of action remained unclear. Although inflammatory and oxidative stress pathways in CRS have been intensively studied, the roles of ferroptosis and anoikis, which may be activated by these pathways, have received little attention.

**Methods:**

First, the active components of WPTLD were obtained through the TCMSP and Herb databases, and then identified using UHPLC-HRMS. Subsequently, target prediction of the identified components was carried out via the SwissTargetPrediction platform. While CRS-related targets were retrieved from GEO, GeneCards, and PharmGKB. A gene library of ferroptosis- and anoikis-associated targets was established. Tissue-specific mRNA expression profiles were analyzed via BioGPS. Subsequently, protein-protein interaction (PPI) networks were constructed to identify core targets, followed by Gene Ontology (GO) and Kyoto Encyclopedia of Genes and Genomes (KEGG) enrichment analyses using Metascape. Finally, molecular docking assessed binding affinities between active components and core targets, with top-ranked complexes undergoing molecular dynamics (MD) simulations.

**Results:**

Fifteen bioactive components and 39 component-disease interaction targets were identified, predominantly localized in kidney, thymus, lung, adipocytes, adrenal gland, and heart tissues. Topological analysis of PPI networks revealed eight core targets, including ferroptosis-/anoikis-associated SIRT1, PTGS2, and PRKCA. KEGG analysis highlighted critical pathways such as AMPK and PI3K-Akt signaling. Notably, molecular docking and MD simulations demonstrated stable binding between active compounds and core targets.

**Conclusion:**

This study systematically deciphers WPTLD’s anti-CRS mechanisms via targeting ferroptosis- and anoikis-related genes through multi-pathway modulation. These findings not only clarify the pathological roles of ferroptosis and anoikis in CRS but also provide a computational framework for developing therapeutic strategies.

## 1 Introduction

Cardiorenal syndrome (CRS) is a clinical syndrome characterized by acute or chronic injury to one organ (heart or kidney) resulting from acute or chronic dysfunction of the other (Ronco et al., 2010). Over 60% of patients with acute decompensated heart failure exhibit coexisting chronic kidney disease (CKD) (McCallum and Sarnak, 2023). Globally, the prevalence of CKD continues to rise, serving as an independent risk factor for cardiovascular diseases (CVDs) such as coronary artery disease and congestive heart failure. Notably, CKD patients demonstrate higher CVD prevalence, with CVD incidence and mortality rates positively correlating with CKD severity (Bikbov et al., 2020; Bagshaw et al., 2010). Despite these associations, the pathophysiological mechanisms underlying CRS remain incompletely understood. Current evidence suggests that CRS pathogenesis may involve hemodynamic alterations, neurohumoral dysregulation, inflammation, oxidative stress, endothelial dysfunction, and iron metabolism abnormalities (Kim et al., 2023; Obi et al., 2016). While inflammatory and oxidative stress mechanisms in CRS are well-established (Rangaswami et al., 2019) and known to trigger ferroptosis (Chen et al., 2023), research specifically investigating ferroptosis in CRS remains limited.

Ferroptosis, an iron-dependent regulated cell death driven by lethal lipid peroxidation accumulation, has emerged as a critical player in cardiovascular and renal pathophysiology. Inhibition of ferroptosis reduces cardiomyocyte death, alleviates heart failure (HF) symptoms, and delays HF progression (Chen et al., 2024). Moreover, ferroptosis significantly contributes to HF and CKD progression, representing a promising therapeutic target (Wang J. et al., 2022; Wang K. et al., 2022). Another form of cell death, anoikis, which is a type of programmed cell death occurring when cells lose contact with the extracellular matrix (ECM), has garnered increasing attention and is widely applied in cancer research. Although initially studied in cancer, anoikis was implicated in cardiovascular diseases (CVDs) as early as 2003 (Michel, 2003). Recent cellular experiments further demonstrate that modulating anoikis suppresses renal fibrosis (Liu et al., 2022). Previous studies have shown that during ferroptosis, lipid peroxidation can directly disrupt cell membrane structure, oxidize ECM components, and promote matrix degradation, thereby affecting cell-ECM interactions and potentially inducing anoikis (Dixon et al., 2012). Thus, this study focuses on the mechanisms of ferroptosis and anoikis, aiming to uncover their unknown roles in CRS and establish new directions for researching the pathogenesis of CRS.

Clinically, diuretics remain the primary strategy for managing volume overload in CRS. However, diuretic resistance frequently develops in advanced CRS (Freda et al., 2011), complicating treatment. Concurrent cardiac and renal dysfunction substantially increases mortality, complication rates, and healthcare costs, leading to poor prognoses (Forman et al., 2004). These challenges underscore the urgent need for novel therapeutic approaches. Traditional Chinese Medicine (TCM), guided by the holistic concept of systemic organ regulation and balance, aligns well with the multifactorial pathogenesis of CRS.

The Wenpitongluo Decoction (WPTLD), derived from two classical TCM formulas, Linggui Zhugan Decoction (LGZGD) and Huangqi Chifeng Decoction (HQCFD, incorporates additional components (Shenqu and Yimucao). These medicines come from various parts of different plants and have edible and medicinal properties. LGZGD exhibits anti-inflammatory, antioxidant, and cardioprotective properties, demonstrating therapeutic potential in HF (Wang and Huang, 2024; Sun S. et al., 2022). Pharmacological studies reveal that LGZGD contains multiple bioactive components capable of treating nephrotic syndrome via multi-target mechanisms (Shi et al., 2023). Modified HQCFD formulations regulate apoptosis, suppress mesangial cell inflammatory proliferation, and mitigate chronic glomerulonephritis progression (Ma et al., 2023). Furthermore, HQCFD derivatives ameliorate podocyte injury, reduce proteinuria, and alleviate renal fibrosis and glomerulosclerosis (Zhao et al., 2022; Zhao et al., 2024). Clinical observations indicate promising efficacy of WPTLD in CRS management.

**FIGURE 1:**
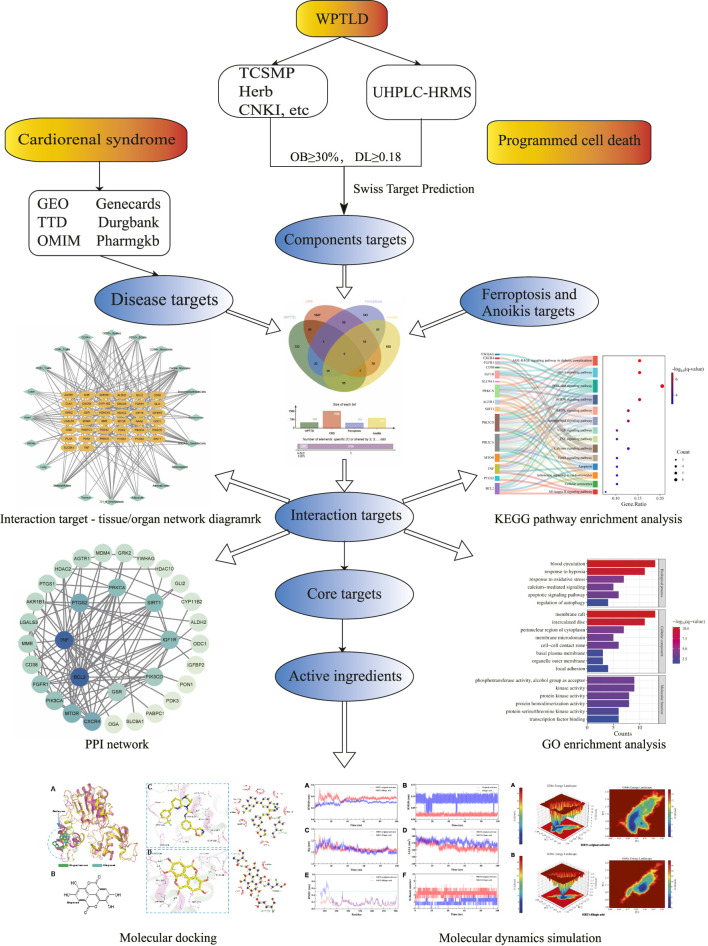
Flow chart of the study.

Building on this evidence, our study employs computational biology strategies, including network pharmacology, molecular docking, and molecular dynamics simulations, to investigate the molecular mechanisms of WPTLD in CRS treatment, with a focus on ferroptosis and anoikis pathways. This integrative approach aims to elucidate novel pathological mechanisms and therapeutic targets for CRS. The research workflow is illustrated in Figure 1.

## 2 Materials and methods

### 2.1 Screening of WPTLD active components and targets

WPTLD comprises Fuling (FL; Poria), Guizhi (GZ; Cinnamomi Ramulus), Baizhu (BZ; Atractylodis Macrocephalae Rhizoma), Gancao (GC; Glycyrrhizae Radix), Huangqi (HQ; Astragali Radix), Chishao (CS; Paeoniae Radix Rubra), Fangfeng (FF; Saposhnikoviae Radix), Yimucao (YMC; Leonuri Herba), and Chaoshenqu (SQ; Massa Medicata Fermentata). Chemical constituents of these herbs were retrieved from the Traditional Chinese Medicine Systems Pharmacology Database (TCMSP). Components unavailable in TCMSP were supplemented via the Herb Database (http://herb.ac.cn/) and literature mining. Compounds were screened based on oral bioavailability (OB) of ≥30% and drug-likeness (DL) of ≥0.18. Those without predicted target proteins were excluded.

### 2.2 UHPLC-HRMS analysis

All herbal materials were obtained from Guang’anmen Hospital, China Academy of Chinese Medical Sciences. After 30-min soaking, herbs were decocted twice for 1 h each in 8 volumes of water at 100°C under atmospheric pressure. The resulting decoctions were combined and concentrated to a density of 2.5 g/mL. WPTLD components were analyzed using ultra-performance liquid chromatography coupled with high-resolution mass spectrometry (UHPLC-HRMS; ACQUITY UPLC I-Class HF system, Waters Corporation) equipped with an ACQUITY UPLC HSS T3 column (100 mm × 2.1 mm, 1.8 µm; Waters Corporation) and a Thermo Orbitrap QE mass spectrometer (Thermo Fisher Scientific). For detailed information, please refer to the Supplementary Material.

### 2.3 Construction of target libraries

Target prediction for mass spectrometry-identified active components was performed using the SwissTargetPrediction platform to obtain WPTLD targets. Two datasets (GSE66494 and GSE21610) were retrieved from the GEO database using the keyword “cardiorenal syndrome.” mRNA expression profiles were analyzed via multi-chip joint analysis with the R limma package, identifying differentially expressed genes (DEGs) using thresholds of |log2FC| > 1 and P < 0.05. Additionally, CRS-related disease targets were collected from five databases: GeneCards (http://www.genecards.org/), OMIM (http://www.omim.org/), Therapeutic Target Database (TTD; http://db.idrblab.net/ttd/), DrugBank (https://go.drugbank.com/), and PharmGKB (https://www.pharmgkb.org/). The retrieved targets were merged and deduplicated to establish a CRS target library. Ferroptosis-related genes were downloaded from the FerrDb database (http://www.zhounan.org/ferrdb/current/), while anoikis-associated genes were obtained by searching “anoikis” in GeneCards. Corresponding target libraries were constructed for ferroptosis and anoikis.

### 2.4 Venn diagram visualization

The intersection of WPTLD component targets (identified via mass spectrometry), CRS targets, ferroptosis genes, and anoikis genes was analyzed using Venny 2.1.0 (http://bioinfo.cnb.csic.es/tools/venny/). This generated component-disease interaction targets and overlapping targets between ferroptosis/anoikis genes, visualized through Venn diagrams.

### 2.5 GO and KEGG enrichment analyses

Interaction targets were uploaded to the Metascape platform with species set to *Homo sapiens* for Gene Ontology (GO) and Kyoto Encyclopedia of Genes and Genomes (KEGG) pathway enrichment analyses. GO analysis included biological processes (BP), molecular functions (MF), and cellular components (CC), with significance thresholds of false discovery rate (FDR) < 0.05 and *P* < 0.05. Results were visualized using R software (version 3.4.1).

### 2.6 PPI network and “component-target-pathway” network construction

Interaction targets were imported into the STRING database (https://www.string-db.org/) to construct a protein-protein interaction (PPI) network model, with species restricted to *Homo sapiens* and interaction confidence set to “highest confidence” (>0.9). The PPI network was visualized using Cytoscape 3.9.0. Network topology parameters—including degree, betweenness, and closeness centrality—were analyzed via the built-in Network Analyzer tool to identify core therapeutic targets of WPTLD for CRS. A “component-target-pathway” network was further constructed by mapping WPTLD components, their targets, and enriched KEGG pathways in Cytoscape 3.9.0. Key bioactive components were screened based on topological parameters.

### 2.7 Tissue-organ network analysis

To explore potential metabolic sites and target organs of WPTLD, mRNA expression levels of interaction targets across tissues were retrieved from the BioGPS database (https://biogps.org). A “target-tissue/organ” network was generated using Cytoscape 3.7.1.

### 2.8 Molecular docking and molecular dynamics (MD) simulations

Molecular docking was performed using AutoDock v4.2.6 and CB-Dock2. Ligand 3D structures were obtained from PubChem (https://pubchem.ncbi.nlm.nih.gov/), while core target protein structures were downloaded from the RCSB PDB (https://www.rcsb.org/). Target proteins were prepared in AutoDock 4.2.6, with docking grids generated via AutoGrid. Docking simulations were executed using AutoDock Vina, and results were visualized in PyMol v2.6 and LigPlot + v2.2.8.

Subsequently, 100 ns molecular dynamics (MD) simulations were performed on the protein-ligand complexes obtained from molecular docking using GROMACS v2022.03 (Van Der Spoel et al., 2005; Abraham et al., 2015). The CHARMM36 force field (Klauda et al., 2010) was employed for the protein system, while the GAFF force field (Özpınar et al., 2010) was assigned to the ligand using AmberTools22. The ligand was hydrogenated and subjected to RESP charge calculation using Gaussian 16 W. The protein-ligand complex was solvated in a TIP3P water model (Nayar et al., 2011) with a minimum distance of ≥1.2 nm between protein atoms and the edge of the cubic water box. System charge neutralization was achieved by adding appropriate numbers of Na^+^ and Cl^−^ ions (concentration: 0.154 M).

Energy minimization (EM) was conducted using the steepest descent algorithm (Donnelly et al., 2021). The system was then gradually heated from 0 K to 300 K under the isothermal-isochoric (NVT) ensemble with solute position restraints, followed by equilibration at 300 K and 1 bar pressure in the isothermal-isobaric (NPT) ensemble. Finally, production MD simulations were conducted for 100 ns with trajectory recording.

The MD trajectories were analyzed for root mean square deviation (RMSD), root mean square fluctuation (RMSF), radius of gyration (Rg), solution accessible surface area (SASA), and hydrogen bond (H-bond) formation. Gibbs free energy was calculated using the built-in GROMACS utilities “g_sham” and “xpm2txt.py” based on RMSD and Rg values. Additionally, binding free energy was estimated using the “MMPBSA.py v.16.0” script (Genheden and Ryde, 2015) through MM/PBSA calculations.

## 3 Results

### 3.1 Active components from TCMSP database

By searching the TCMSP database for the active ingredients of WPTLD, a total of 166 active ingredients were retrieved. Among them, there were 6 Poria, 7 Gui Zhi, 4 Fried Atractylodes, 88 Glycyrrhiza, 20 Raw Astragalus, 15 Paeonia lactiflora, 18 Saposhnikovia divaricata, and 8 Yi Mu Cao; and 36 active ingredients were screened for the active ingredients of the Fried Shen Qu through the Herb database as well as literature search. After screening, 144 active ingredients were retained (Supplementary Table S6).

### 3.2 UHPLC-HRMS analysis

**FIGURE 2:**
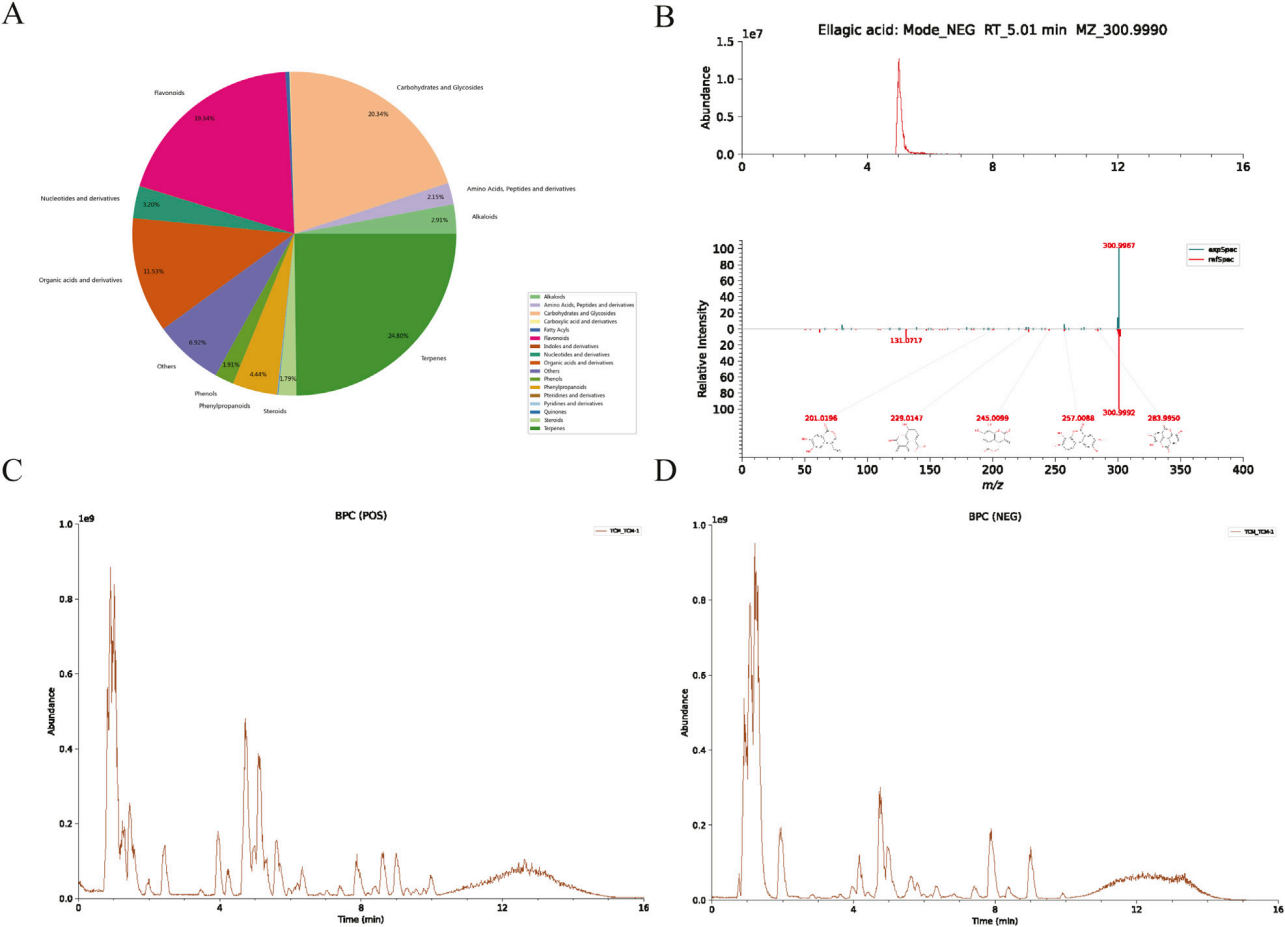
UHPLC-HRMS analysis results. **(A)** Content distribution and classification of herbal components; **(B)** EIC (Extracted Ion Chromatogram) of ellagic acid and its MS/MS spectrum compared with the LuMet-TCM standard library; **(C)** BPC (Base Peak Chromatogram) diagram in the positive ion mode; **(D)** BPC diagram in the negative ion mode.

UHPLC-HRMS, covering both mass spectrometry and chromatography, was used to analyze WPTLD and detected 960 compounds (Supplementary Table S5). An intersection was taken with the 144 active components from “Section 3.1”, and 15 effective bioactive components were identified by ensuring OB ≥ 30% and DL ≥ 0.18 (Table 1). Specifically, the categories of compounds detected by UHPLC-HRMS are shown in Figure 2A; the chromatograms of the compounds are in Figure 2B; and the mass spectra are presented in Figures 2C,D.

**TABLE 1:** Active ingredient information table.

PubChem CID	Metabolites	OB%	DL	SMILES
5281605	Baicalein	33.52	0.21	C1(O)=CC2OC(C3C=CC=CC=3)=CC(=O)C=2C(O)=C1O
5281855	Ellagic acid	43.06	0.43	OC1=C(O)C2=C3C(=C1)C(=O)OC1=C3C(=CC(O)=C1O)C(=O)O2
124052	Glabridin	53.25	0.47	C12OC(C)(C)C=CC1=C1OC[C@@]([H])(C3C(O)=CC(O)=CC=3)CC1=CC=2
5281619	Glepidotin A	44.72	0.35	C1C=C(C2=C(O)C(=O)C3C(O)=CC(O)=C(C/C=C(\C)/C)C=3O2)C=CC=1
480859	Glyasperin C	45.56	0.40	CC(=CCC1=C(C2=C(C=C1O)OCC(C2)C3=C(C=C(C=C3)O)O)OC)C
480787	Glycyrin	52.61	0.47	C1(OC)C(C/C=C(\C)/C)=C(OC)C2C=C(C3=CC=C(O)C=C3O)C(=O)OC=2C=1
5318585	Isolicoflavonol	45.17	0.42	C1(O)=CC2OC(C3C=CC(O)=C(C/C=C(\C)/C)C=3)=C(O)C(=O)C=2C(O)=C1
5281654	Isorhamnetin	49.6	0.31	C1(O)C=C2OC(C3=CC=C(O)C(OC)=C3)=C(O)C(=O)C2=C(O)C=1
5318679	Isotrifoliol	31.94	0.42	COC1=C2C3=C(C4=C(O3)C=C(O)C=C4)C(=O)OC2=CC(O)=C1
5280863	Kaempferol	41.88	0.24	C1(O)C=C2OC(C3=CC=C(O)C=C3)=C(O)C(=O)C2=C(O)C=1
5318999	Licochalcone B	76.76	0.19	C1(O)=CC=C(C(=O)/C=C/C2C=CC(O)=C(O)C=2OC)C=C1
5319013	Licoricone	63.58	0.47	C1(O)=CC2OC=C(C3C(O)=CC(OC)=C(C/C=C(/C)\C)C=3OC)C(=O)C=2C=C1
336327	Medicarpin	49.22	0.34	C1(O)=CC2OC[C@@]3([H])C4C=CC(OC)=CC=4O[C@@]3([H])C=2C=C1
442534	Paeoniflorin	53.87	0.79	CC12CC3(O)OC(O1)C1(COC(=O)C4=CC=CC=C4)C3CC21OC1OC(CO)C(O)C(O)C1O
5481948	Semilicoisoflavone B	48.78	0.55	C1(O)C=C(O)C2C(=O)C(C3=CC(O)=C4OC(C)(C)C=CC4=C3)=COC=2C=1

### 3.3 Network pharmacology visualization results

**FIGURE 3:**
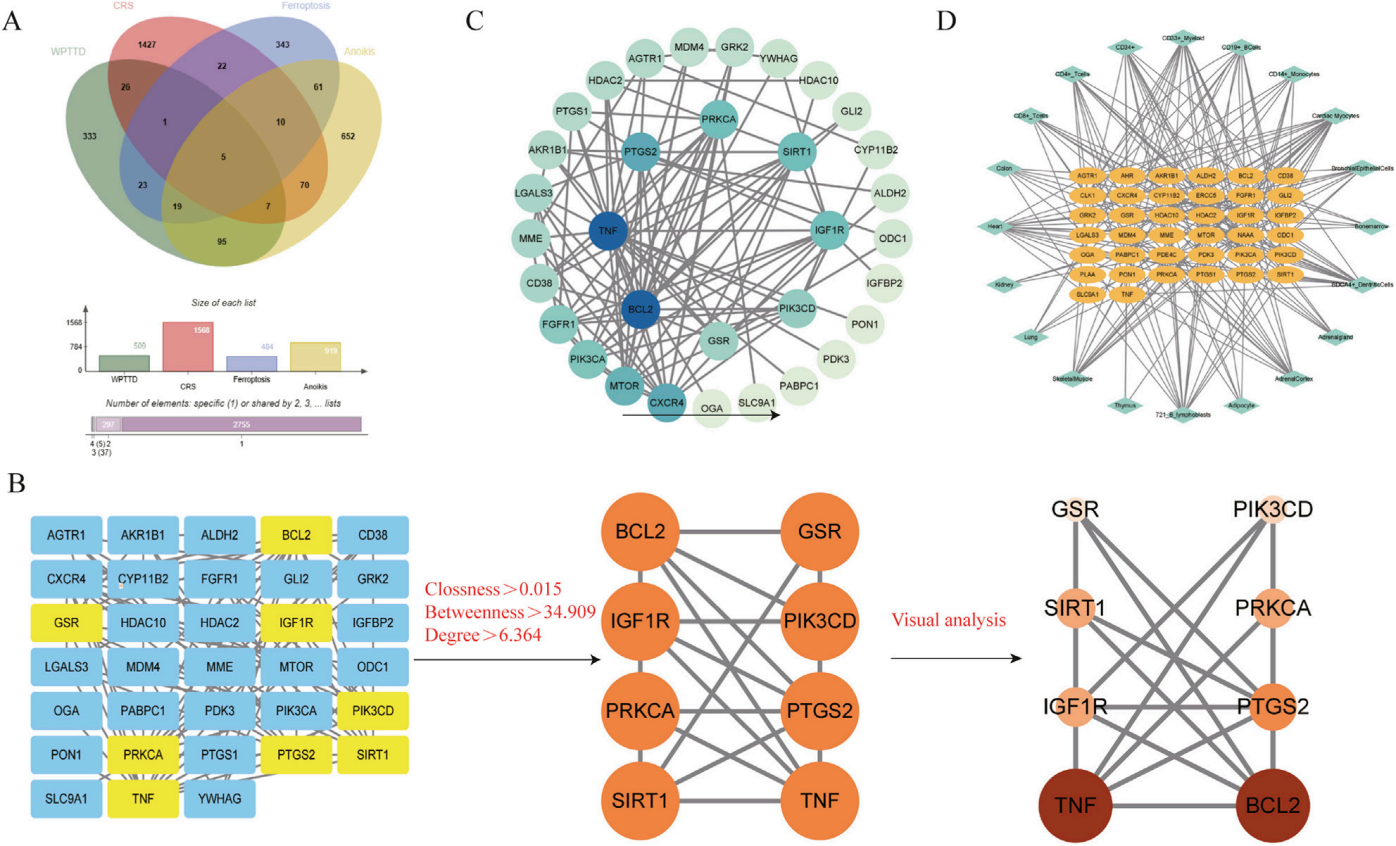
WPTLD target screening and tissue network. **(A)** Component - disease target Venn diagram. **(B,C)** PPI network diagrams. **(D)** Interaction target - tissue/organ network diagram.

Target prediction for the identified components via SwissTargetPrediction yielded 509 targets after deduplication. Integrated analysis of disease-related genes from multiple sources identified 1568 CRS targets, 484 ferroptosis-related genes, and 919 anoikis-associated genes. Venn diagram analysis (Figure 3A) revealed 39 component-disease interaction targets (Table 2).

**TABLE 2:** Venn diagram component - disease interaction targets.

No.	Gene	Class
1	AKR1B1	
2	PTGS2	*△
3	CD38	
4	AHR	
5	IGF1R	△
6	SIRT1	*△
7	GSR	
8	ALDH2	
9	PON1	
10	ODC1	
11	CYP11B2	
12	HDAC10	
13	HDAC2	
14	BCL2	△
15	PIK3CD	
16	NAAA	
17	PIK3CA	*△
18	MTOR	*△
19	MDM4	*
20	PDE4C	
21	IGFBP2	
22	CXCR4	△
23	CLK1	
24	GLI2	△
25	YWHAG	
26	PTGS1	
27	GRK2	
28	SLC9A1	
29	AGTR1	
30	PDK3	
31	ERCC5	
32	PRKCA	*△
33	FGFR1	△
34	PLAA	
35	LGALS3	△
36	PABPC1	
37	OGA	
38	MME	
39	TNF	△

* , Ferroptosis - related genes. △, Anoikis - related genes.

**FIGURE 4:**
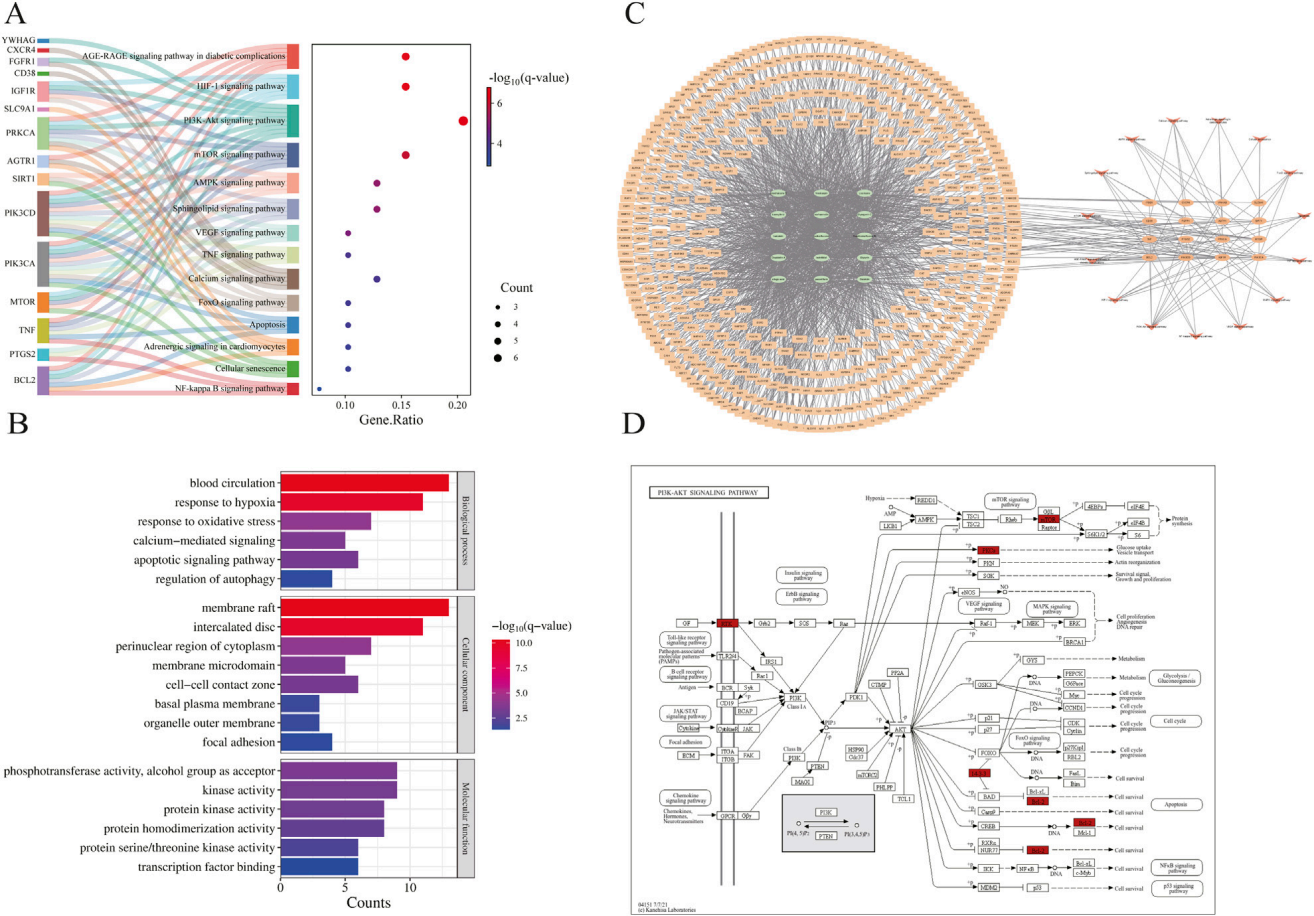
Enrichment analysis and WPTLD component screening. **(A)** KEGG pathway enrichment analysis. **(B)** GO enrichment analysis. **(C)** Component - target - pathway network. **(D)** PI3K - Akt signaling pathway.

The PPI network constructed using STRING (Figure 3C) comprised 33 nodes and 105 edges. Topological analysis based on median degree centrality (DC: 6.364), closeness centrality (CC: 0.015), and betweenness centrality (BC: 34.909) identified 8 core targets (Figure 3B), including ferroptosis-/anoikis-associated SIRT1, PTGS2, and PRKCA. YWHAG was excluded due to unavailable BioGPS data, leaving 38 interaction targets for tissue mapping. The “target-tissue/organ” network demonstrated predominant localization in kidney, thymus, lung, adipocytes, adrenal gland, and heart tissues (Figure 3D). Enrichment analysis was conducted through the Metascape platform, and the graphs were drawn using the adjusted P value (q-value). KEGG pathway was enriched in PI3K-Akt signaling pathway, AMPK signaling pathway, Apoptosis and other signaling pathways (Figure 4A); GO enrichment analysis showed that Biological Processes were enriched in response to oxygen levels, apoptocic signaling pathway, etc., Cellular Component was enriched in membrane raft, intercalated disc, and Molecular Functions was enriched in protein kinase activity, etc. (Figure 4B). The “component-target-pathway” network (Figure 4C) prioritized seven bioactive components: paeoniflorin, kaempferol, isorhamnetin, licoricone, glabridin, ellagic acid, and baicalein. KEGG pathway mapping further annotated core targets within signaling cascades (Figure 4D).

### 3.4 Molecular docking and molecular dynamics simulation

**FIGURE 5:**
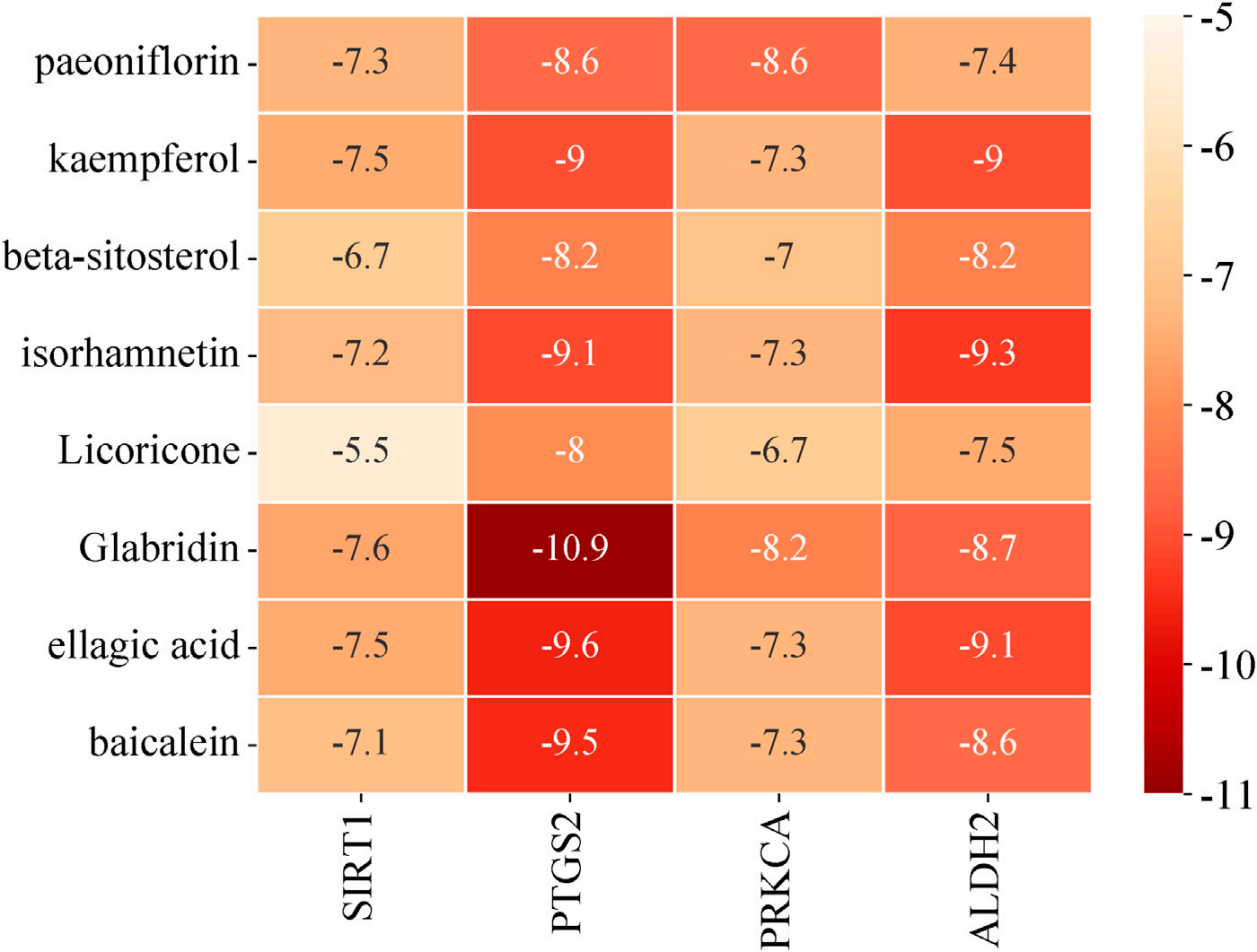
Molecular docking result heatmap.

The major bioactive components selected were paeoniflorin, kaempferol, isorhamnetin, licoricone, glabridin, ellagic acid, and baicalein. Molecular docking verification was performed using the intersection targets of ferroptosis and anoikis, namely, SIRT1 (PDB ID: 4ZZI), PTGS2 (PDB ID: 5F19), and PRKCA (PDB ID: 4DNL). The results are shown in Figure 5. A binding energy of less than 0 kcal/mol indicates that the combination can occur spontaneously, and the lower the binding energy, the stronger the binding ability. In this study, the binding energy between the predicted major bioactive components and the core targets ranged from −5.5 kcal/mol to −10.9 kcal/mol, indicating good binding ability.

**FIGURE 6:**
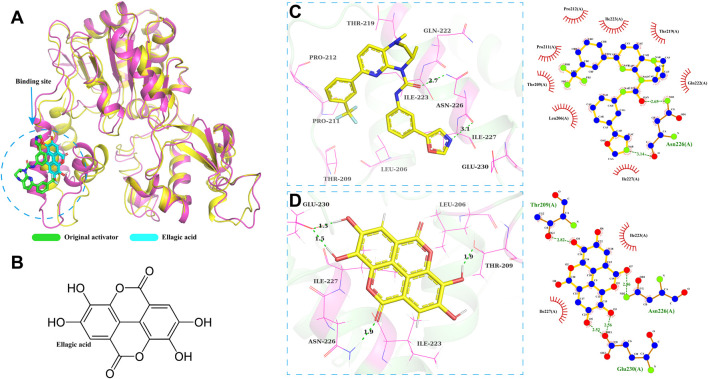
SIRT1 - Ellagic acid molecular docking model. **(A)** Docking model of Ellagic acid and SIRT1. **(B)** 2D structure of Ellagic acid. **(C,D)** Schematic diagrams of the docking results.

The molecular docking model of SIRT1-ellagic acid (Figure 6) shows that the binding site of SIRT1-ellagic acid on SIRT1 is on the original activator. Therefore, in this study, 100 ns of MD simulation analysis was carried out on the SIRT1-ellagic acid and SIRT1-original activator complex systems, including root mean square deviation (RMSD), root mean square fluctuation (RMSF), radius of gyration (Rg), solution accessible surface area (SASA), and statistical analysis of hydrogen bond changes throughout the process, to study the dynamic properties of the molecular docking. As shown in the molecular docking model of ellagic acid and SIRT1 in Figure 6A, the optimal docking conformation of ellagic acid and SIRT1 overlaps with that of the original activator and is closer to the protein’s β - sheet region. From the 2D structure of ellagic acid (Figure 6B), it can be found that ellagic acid has 4 hydroxyl groups and 2 lactone ring structures. We speculate that these groups enable ellagic acid to form more stable interactions with the β - sheet region. Subsequently, a visual analysis of the two complex systems was carried out. From the 3D and 2D diagrams, it can be seen that the original activator only forms hydrogen bonds with the Asn226 residue of SIRT1 and has hydrophobic interactions with surrounding amino acids (Figure 6C). In contrast, ellagic acid can form 1 hydrogen bond with Thr209 of SIRT1, 1 hydrogen bond with Asn226, and 2 hydrogen bonds with Glu230, as well as hydrophobic interactions with surrounding amino acids. Therefore, we consider that ellagic acid can form a more stable complex with SIRT1 than the original activator.

**FIGURE 7:**
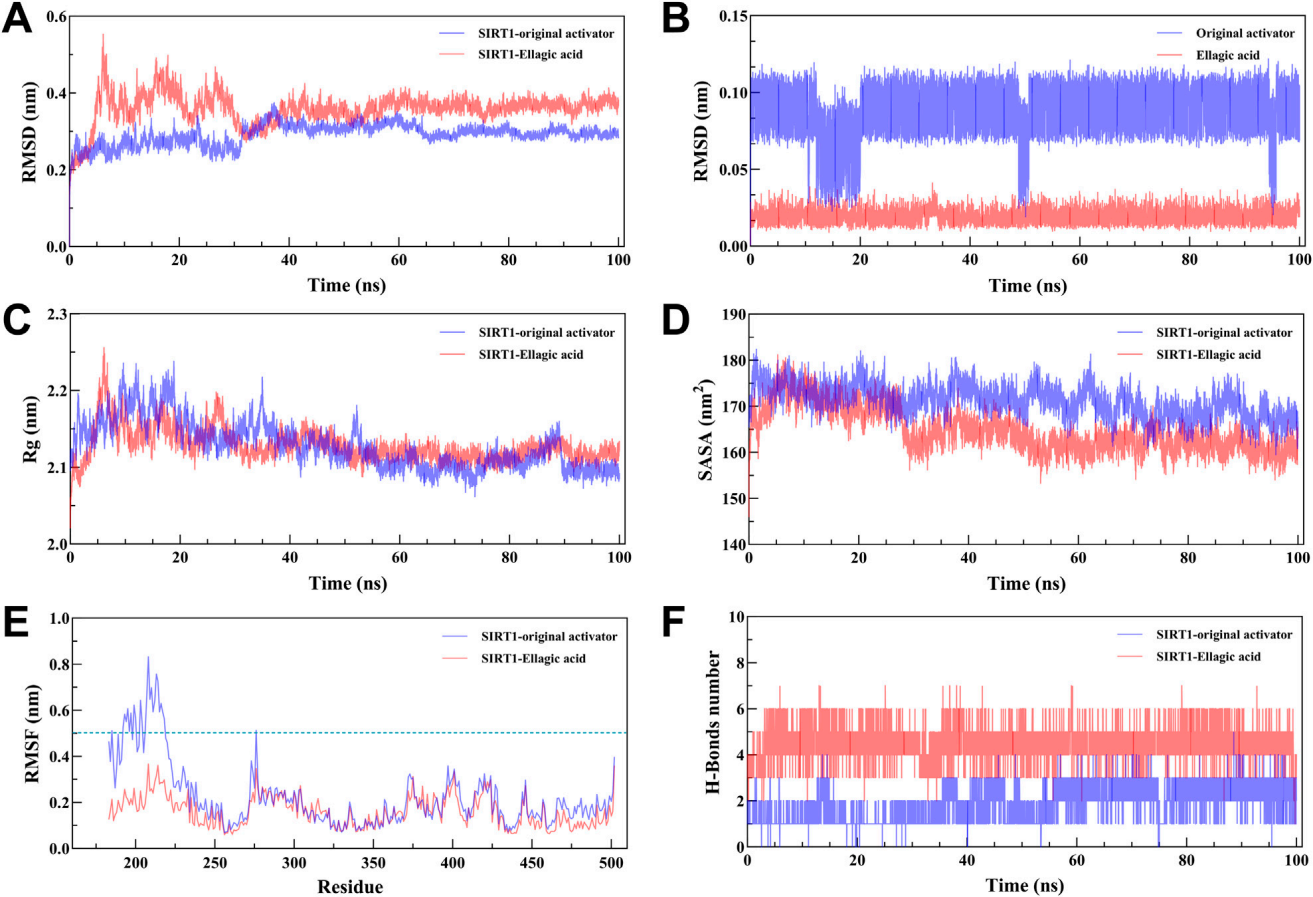
100 ns MD simulation analysis of the SIRT1 - Ellagic acid complex. **(A)** RMSD curve of the complex. **(B)** RMSD curve of the small molecule. **(C)** Rg curve of the complex. **(D)** SASA curve of the complex. **(E)** RMSF curve of SIRT1. **(F)** Hydrogen bond change curve of the complex.

**FIGURE 8:**
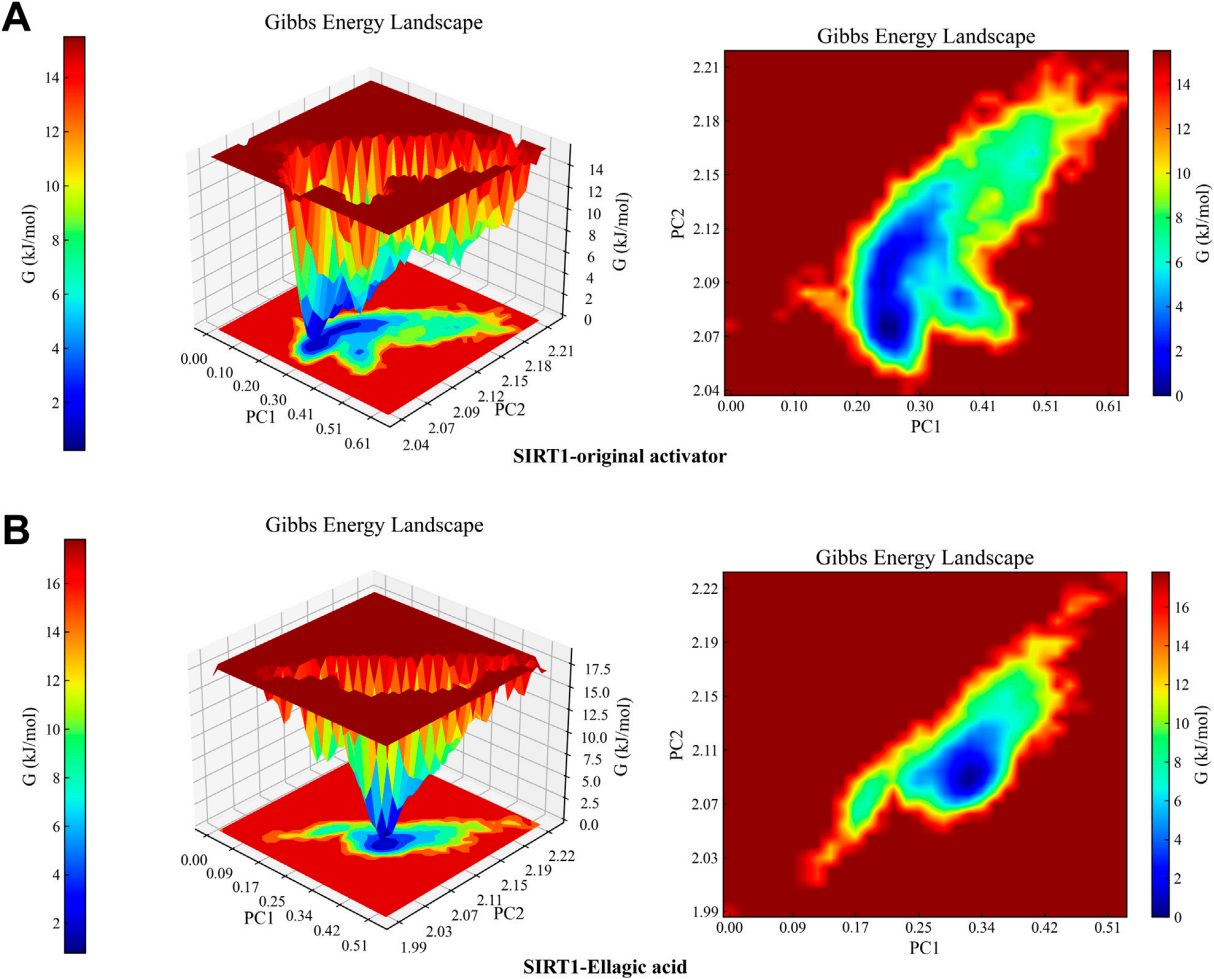
Complex Gibbs free energy analysis. **(A)** 3D and 2D free energy landscapes of the SIRT1 - original activator complex. **(B)** 3D and 2D free energy landscapes of the SIRT1 - Ellagic acid complex. Blue - and purple - shaded areas indicate that the stable conformation of the complex can be mapped at lower energy within the minimum free energy zone. Weak or unstable interactions lead to multiple, rough clusters in the free energy landscape, while strong, stable interactions form single, smooth clusters.

MD results show that the RMSD curves (Figure 7A) of the two complex systems gradually tend towards equilibrium after 40 ns, and the RMSD fluctuation range of both is within 1 nm, indicating that both the original activator and ellagic acid can form stable complex systems with SIRT1. In addition, to study the effect of small molecule binding on the flexibility of protein amino acid residues, the RMSF values of the amino acids of SIRT1 were calculated. As shown in the RMSF results (Figure 7B), during the 100 ns simulation, the original agonist had 3 severe fluctuations, while ellagic acid maintained stability throughout the MD process, indicating that the small molecule ellagic acid can bind continuously and stably to SIRT1. Notably, compared with the original activator system, the binding of ellagic acid significantly reduces the flexibility of amino acid residues in this region. This result further indicates that ellagic acid can form more stable interactions with amino acid residues in this region than the original activator, thereby reducing the fluctuation of amino acids in this region and helping ellagic acid maintain continuous stability (Figure 7E). Rg analysis shows that the Rg curves of the two complex systems have the same trend and overlap, indicating that the changes in the tightness of the SIRT1 protein during the simulation are the same (Figure 7C). SASA analysis shows that the binding of ellagic acid results in a lower SASA value of the protein than the original activator system after 30 ns, suggesting that ellagic acid forms more hydrophobic interactions with SIRT1, reducing the hydrophilicity of the protein (Figure 7D). As shown in Figure 7F, the number of hydrogen bonds in the original activator system ranges from 1 to 5, while that in the ellagic acid system ranges from 4 to 7. Ellagic acid can form more hydrogen bonds with SIRT1 than the original activator, which is beneficial for the formation of a more stable complex system between ellagic acid and SIRT1. The Gibbs free energy landscape describes the stability of the receptor - ligand complex. As shown in Figure 8A, the 3D topography of the Gibbs free energy of the SIRT1 - original activator complex is relatively rough, and the 2D diagram shows the presence of 2 minimum energy zones. In contrast, the 3D topography of the Gibbs free energy of the SIRT1 - ellagic acid complex forms a nearly single and smooth energy cluster, consistent with the 2D diagram (Figure 8B). This result indicates that among the selected compounds, ellagic acid has the most stable dynamic conformation during the MD simulation, which is consistent with the MD results of the RMSD analysis. According to the MD calculation results, the total binding free energy of the SIRT1 - original activator complex is −7.71 kcal/mol, while that of the SIRT1 - ellagic acid complex is −10.89 kcal/mol (Table 3).

**TABLE 3:** Average binding free energy (kcal/mol) of the two complexes calculated by the MM/PBSA method.

Energy contributions	SIRT1-original activator	SIRT1-ellagic acid
ΔVDWAALS	−8.31	−6.16
ΔE_elec_	−3.23	−10.92
ΔE_GB_	4.94	9.27
ΔE_surf_	−1.11	−0.69
ΔG_gas_	−11.54	−19.47
ΔG_solvation_	3.83	8.58
ΔTotal	−7.71	−10.89

## 4 Discussion

CRS involves crosstalk between the heart and kidneys, with both pathological links and causal relationships. Its treatment remains exploratory. Many heart failure (HF) medications may negatively impact kidney function, and vice-versa. For instance, diuretics can reduce cardiac load but may cause hypovolemia, decreased renal perfusion, and pre - renal AKI if over-used. Similarly, high-dose intravenous fluid to correct pre-renal AKI (e.g., from hypovolemia or shock) can worsen HF by increasing cardiac preload. These situations complicate CRS treatment and management. In contrast, traditional Chinese medicine (TCM) formulas can simultaneously target the heart and kidneys. With growing research and development in TCM formulas, more evidence-based studies have confirmed their efficacy and safety, marking a new direction for clinical research. Our study identified the major bioactive components of WPTLD via UHPLC-HRMS as paeoniflorin, kaempferol, isorhamnetin, licoricone, glabridin, ellagic acid, and baicalein. The core targets associated with ferroptosis and anoikis were SIRT1, PTGS2, and PRKCA. BioGPS database analysis showed these targets are highly expressed in multiple organs. Molecular docking and MD simulations validated the good binding ability between active molecules and targets.

In the study of cardiorenal disease treatment, flavonoids have been widely studied for their diverse biological activities. Previous research has shown that some flavonoids, such as kaempferol and isorhamnetin, have significant antioxidant, anti-inflammatory and cardioprotective effects (Sun S. et al., 2022; Dong et al., 2015). Moreover, recent studies have found that flavonoids can also inhibit ferroptosis and regulate apoptosis through multiple pathways, thereby exerting cardioprotective properties (Xu et al., 2024). This study not only reveals the potential of these compounds in CRS treatment but also explores their specific mechanisms in regulating ferroptosis and apoptosis, offering key insights for their precise application in disease intervention. Cell experiments have confirmed kaempferol as a key component of LGZGD, effective in treating HF (Sun S. et al., 2022). WPTLD, derived from LGZGD, has been shown by UHPLC-HRMS to retain the active component kaempferol, indirectly proving its therapeutic effect on HF. Furthermore, this study has demonstrated that the active components function by targeting core targets such as SIRT1. This is consistent with prior research findings. Wang A discovered that kaempferol can effectively reduce the expression of pro-inflammatory factors and inhibit the occurrence of oxidative stress and ferroptosis by activating SIRT1, including the HMGB1/TLR4/NF-κB and NRF2/SLC7A11/GPX4 pathways (Wang et al., 2025).

Previous study have shown that baicalein can enhance the ability of cells to resist ferroptosis, making it a potential therapeutic agent for ferroptosis - related tissue damage (Xie et al., 2016). For example, Wang IC (Wang IC. et al., 2023) and Fan ZY (Fan et al., 2021) found that baicalein protected cardiomyocytes from ferroptosis induced by ferroptosis inducers and ischemia/reperfusion (I/R). Liang GQ discovered that baicalein improved renal function, inhibited renal ferroptosis, and slowed renal fibrosis (Qiang et al., 2024). These findings highlight baicalein’s promise in cardiorenal disease therapy. Animal and cell experiments in an AKI model showed baicalein downregulated Fe^2+^, MDA, and PTGS2, while upregulating SCL7A11, GPX4, and GSH, exerting ferroptosis - inhibiting effects. This was achieved by enhancing SIRT1 expression to promote p53 deacetylation (Yu et al., 2023). This was consistent with the core target of action identified by this study through computational biology. Moreover, previous studies have shown that baicalein can exert cardioprotective effects by inhibiting apoptosis and inflammation (Qu et al., 2016). Anoikis was a subtype of apoptosis, which suggests that baicalein might suppress anoikis. In this study, we discovered that baicalein exerts therapeutic effects on CRS by targeting anoikis - related targets, advancing its research in anoikis.

Ellagic acid was a natural polyphenolic compound. Besides its strong antioxidant activity, in an experiment on a rat model of kidney injury, it was found that after intervention with ellagic acid, the levels of PTGS2 and MDA in rat serum decreased, while the activities of SOD and GSH significantly increased. The renal tissue structure was improved, indicating that ellagic acid also has an iron - death - improving effect. Meanwhile, it was observed that ellagic acid can inhibit apoptosis and autophagy pathways by reducing the expression of LC3B (Bhattacharjee et al., 2021). This finding has been confirmed by other scholars. Research showed that ellagic acid can regulate cellular iron metabolism, boost free-iron excretion, activate the Nrf2/keap1 pathway, and increase GPX4 synthesis, thereby alleviating oxidative stress and ferroptosis (Yang et al., 2024). This expands the research on ellagic acid, a natural polyphenol, in cardioprotection and offers new directions for its use in treating CRS and other diseases. Our study indicates that in CRS, ellagic acid may protect cardiac and renal functions via antiferroptotic and anoikis - inhibiting effects.

Studies have shown that SIRT1 was a core target in ferroptosis, anoikis, and CRS. As a member of the Sirtuins (SIRTs) family, SIRT1 was widely distributed in cells and has strong deacetylase activity. Research has indicated that the activation or overexpression of SIRT1 can deacetylate p53. Since p53 can directly inhibit the GPX4 pathway and, under ROS, indirectly enhance the lipoxygenase (LOX) family’s function by inhibiting SLC7A11 (Jiang et al., 2015), SIRT1’s deacetylation of p53 blocks this process, thereby inhibiting ferroptosis (De Angelis et al., 2015; Chen et al., 2022). These studies have revealed another function of SIRT1 and a novel therapeutic strategy: inhibiting ferroptosis by activating SIRT1. In this study, molecular docking and MD simulations showed that ellagic acid, an active component of WPTLD, can stably bind to the activation site of SIRT1. Notably, previous research has confirmed that ellagic acid can form a stable bond with the SIRT1 activation site. It has also been shown that SIRT1 activation leads to the deacetylation and activation of Nuclear factor E2-related factor 2 (NRF2), thereby regulating the cellular antioxidant response (Bai et al., 2025). The activation of NRF2 upregulates antioxidants like GPX4 and GCLC. These enzymes scavenge lipid peroxides, prevent the build-up of lipid peroxidation products like MDA, and inhibit ferroptosis (Shi and Ning, 2025). In a myocardial infarction study, the activation of the SIRT1/AMPK signaling pathway following traditional Chinese medicine intervention promoted mitophagy, which in turn suppressed oxidative stress and inflammatory responses in cardiomyocytes, thus improving cardiac function (Sun X. et al., 2022). AMPK is a key cellular energy sensor that plays an important role in maintaining energy homeostasis and regulating cellular metabolism. Meanwhile, the AMPK signaling pathway has a dual - edged regulation on ferroptosis. On the one hand, AMPK activation can protect cells from ferroptosis by inhibiting lipid peroxidation and enhancing antioxidant capacity. On the other hand, activated AMPK may also promote ferroptosis by regulating specific signaling pathways such as mTOR and SLC7A11 (Wang F. et al., 2023). In conclusion, in the CRS model, SIRT1 may exert therapeutic effects by regulating oxidative stress and ferroptosis through multiple pathways.

Anoikis, a physiological process and special programmed cell death, contributes to tissue and cell homeostasis. In 2003, Michel JB linked Anoikis to CVD, suggesting it may drive cell loss in the cardiovascular system. If the balance between Anoikis and cell healing is disrupted, it can result in abnormal tissue remodeling, such as cardiomyocyte loss in early overloaded left ventricles, progressing to HF. In the ECM, Anoikis, along with inhibited cell adhesion and growth, may be a primary obstacle to cell healing, presenting potential new therapeutic targets (Michel, 2003). This finding expands the study of anoikis and ECM in CVD, but so far, it has not been sufficiently emphasized or explored in mechanism - related research. However, Hong Liu analyzed CKD - related genes and anoikis - associated genes and identified common targets. Six hub genes (LAMC2, NRP1, CDH3, NDRG1, CLDN1, and LAMB3) were found through bioinformatics. Then, four CKD mouse models were set up to verify these targets. RT - qPCR tests showed changes in the expression of hub genes in CKD. It turned out that these anoikis - related genes might serve as potential diagnostic markers for CKD (Liu et al., 2025).

This undoubtedly represents a further advancement in the research on anoikis. The above results indicate the association between anoikis and heart and kidney diseases. After heart and kidney damage, anoikis occurs. This then activates macrophages and Myofibroblast (MyoFb) fibrosis, further destroying the ECM structure, exacerbating the cell adhesion disorder, and promoting more anoikis, forming a vicious cycle. The pathological changes of the kidneys in CRS included renal fibrosis, which was characterized by excessive deposition of ECM. MyoFbs were the main cells that generated ECM. When activated, MyoFbs produced large amounts of ECM, leading to fibrosis. Therefore, inhibiting MyoFb activation was a potential therapeutic strategy for controlling disease progression. Studies have demonstrated that acquired anoikis resistance constitutes a hallmark feature of MyoFb activation. Li XH established an anoikis model in MyoFb via TGF-β1 induction and observed significantly lower anoikis rates in MyoFb compared to human proximal tubular epithelial cells (HK-2). Western blot analysis revealed markedly elevated P-PI3K and P-AKT expression alongside reduced cleaved-caspase3 levels in MyoFb. Subsequent administration of PI3K/AKT pathway inhibitors partially reversed the increased proliferation and decreased apoptosis rates in MyoFb, confirming their anoikis resistance mechanism mediated through the PI3K/Akt signaling pathway. Furthermore, TSSC3 intervention effectively reduced PI3K-P85 and AKT levels in MyoFb, demonstrating that downregulating the PI3K/Akt pathway suppresses both anoikis resistance and pro-fibrotic capacity of renal-derived MyoFb, thereby attenuating renal fibrosis (Liu et al., 2022). These results provide detailed explanations of the pathological role of Anoikis in heart and kidney diseases, as well as the significance of the PI3K/Akt signaling pathway. Therefore, in the early treatment of CRS, the occurrence of Anoikis should be avoided as early as possible, the activation of MyoFb should be inhibited, and the development of renal fibrosis should be blocked; while after the kidneys have already developed fibrosis, the PI3K/Akt pathway can be used to inhibit the resistance to anoikis, promote MyoFb apoptosis, thereby delaying renal fibrosis.

In our prior bioinformatics analysis, we found that WPTLD’s targets were associated with anoikis. Thus, we hypothesized that WPTLD might combat CRS by targeting both ferroptosis and anoikis. Anoikis might have contributed to cardiovascular tissue remodeling, such as myocyte detachment in HF, endothelial denudation, and plaque rupture in atherosclerosis. In these contexts, the intracellular mechanisms of anoikis involve PI3K/Akt-mediated regulation of focal adhesions and integrin-linked kinase activity (Aoudjit and Vuori, 2001; Stupack et al., 2001). The PI3K/Akt signaling pathway was a crucial signaling pathway for cell survival. However, when cells lose contact with the ECM, the activity of the PI3K/Akt signaling pathway was inhibited, leading to cell apoptosis. Therefore, controlling this pathway can regulate apoptosis. Studies showed that activating the PI3K/Akt signaling pathway through stimulation or intervention inhibited apoptosis - related proteins like the Bcl-2 family by phosphorylating downstream targets, thus regulating apoptosis (Taddei et al., 2012).

## 5 Conclusion

In summary, this study employed mass spectrometry to identify active components of WPTLD and integrated network pharmacology, molecular docking, and molecular dynamics (MD) simulations to reveal, for the first time, that WPTLD contains bioactive compounds such as ellagic acid and baicalein. These components synergistically regulate the AMPK and PI3K/Akt signaling pathways via SIRT1/PTGS2/PKCA cross-talk, thereby targeting both ferroptosis and anoikis in cardiac and renal cells to combat CRS. While this work uncovers the potential roles of ferroptosis and anoikis in CRS pathogenesis, offering novel insights into its molecular mechanisms and scientific validation for the modernization of traditional Chinese medicine formulations, it remains limited by the absence of *in vivo* and *in vitro* experimental confirmation. Further investigations are warranted to elucidate the interplay between ferroptosis and anoikis, as well as their spatiotemporal dynamics in CRS progression.

## Data Availability

The original contributions presented in the study are included in the article/Supplementary Material, further inquiries can be directed to the corresponding authors.

## Abbreviations

AKI, Acute kidney injury; BP, Biological processes; CC, Cellular components; CKD, Chronic kidney disease; CRS, Cardiorenal syndrome; CVDs, Cardiovascular diseases; ECM, Extracellular matrix; GO, Gene Ontology; HF, Heart failure; HQCFD, Huangqichifeng Decoction; I/R, Sschemia/reperfusion; KEGG, Kyoto Encyclopedia of Genes and Genomes; LGZGD, Lingguizhugan Decoction; MD, Molecular dynamics; MF, Molecular functions; PPI, Protein-protein interaction; PRKCA, Protein kinase C alpha; PTGS2, Prostaglandin-endoperoxide synthase 2; Rg, Radius of gyration; RMSD, Root mean square deviation; RMSF, Root mean square fluctuation; SASA, Solution accessible surface area; SIRT1, NAD-dependent histone deacetylase sirtuin-1; TCM, Traditional chinese medicine; UHPLC-HRMS, Ultra-performance liquid chromatography coupled with high-resolution mass pectrometry; WPTLD, Wenpitongluo Decoction.

## References

[B1] Abraham M. J. Murtola T. Schulz R. Páll S. Smith J. C. Hess B. (2015). GROMACS: high performance molecular simulations through multi-level parallelism from laptops to supercomputers. SoftwareX 1–2, 19–25. 10.1016/j.softx.2015.06.001

[B2] Aoudjit F. Vuori K. (2001). Matrix attachment regulates Fas-induced apoptosis in endothelial cells: a role for c-flip and implications for anoikis. J. Cell Biol. 152 (3), 633–643. 10.1083/jcb.152.3.633 11157988 PMC2196007

[B3] Bagshaw S. M. Cruz D. N. Aspromonte N. Daliento L. Ronco F. Sheinfeld G. (2010). Epidemiology of cardio-renal syndromes: workgroup statements from the 7th ADQI Consensus Conference. Nephrol. Dial. Transpl. 25 (5), 1406–1416. 10.1093/ndt/gfq066 20185818

[B4] Bai X. Liu Y. Liu J. Guo K. Guan H. (2025). ADSCs-derived exosomes suppress macrophage ferroptosis via the SIRT1/NRF2 signaling axis to alleviate acute lung injury in sepsis. Int. Immunopharmacol. 146, 113914. 10.1016/j.intimp.2024.113914 39732105

[B5] Bhattacharjee A. Kulkarni V. H. Chakraborty M. Habbu P. V. Ray A. (2021). Ellagic acid restored lead-induced nephrotoxicity by anti-inflammatory, anti-apoptotic and free radical scavenging activities. Heliyon 7 (1), e05921. 10.1016/j.heliyon.2021.e05921 33490681 PMC7809373

[B6] Bikbov B. Purcell C. A. Levey A. S. Smith M. Abdoli A. Abebe M. (2020). Global, regional, and national burden of chronic kidney disease, 1990–2017: a systematic analysis for the Global Burden of Disease Study 2017. Lancet 395 (10225), 709–733. 10.1016/s0140-6736(20)30045-3 32061315 PMC7049905

[B7] Chen C. Wang J. Zhang S. Zhu X. Hu J. Liu C. (2024). Epigenetic regulation of diverse regulated cell death modalities in cardiovascular disease: insights into necroptosis, pyroptosis, ferroptosis, and cuproptosis. Redox Biol. 76, 103321. 10.1016/j.redox.2024.103321 39186883 PMC11388786

[B8] Chen H. Lin X. Yi X. Liu X. Yu R. Fan W. (2022). SIRT1-mediated p53 deacetylation inhibits ferroptosis and alleviates heat stress-induced lung epithelial cells injury. Int. J. Hyperth. 39 (1), 977–986. 10.1080/02656736.2022.2094476 35853732

[B9] Chen Y. Fang Z. M. Yi X. Wei X. Jiang D. S. (2023). The interaction between ferroptosis and inflammatory signaling pathways. Cell Death Dis. 14 (3), 205. 10.1038/s41419-023-05716-0 36944609 PMC10030804

[B10] De Angelis A. Piegari E. Cappetta D. Russo R. Esposito G. Ciuffreda L. P. (2015). SIRT1 activation rescues doxorubicin-induced loss of functional competence of human cardiac progenitor cells. Int. J. Cardiol. 189, 30–44. 10.1016/j.ijcard.2015.03.438 25889431

[B11] Dixon S. J. Lemberg K. M. Lamprecht M. R. Skouta R. Zaitsev E. M. Gleason C. E. (2012). Ferroptosis: an iron-dependent form of nonapoptotic cell death. Cell 149 (5), 1060–1072. 10.1016/j.cell.2012.03.042 22632970 PMC3367386

[B12] Dong X. Sun G. B. Luo Y. Sun X. B. Chen S. H. (2015). Protective effect of isorhamnetin on H9C2 cell line against oxidative stress. Chin. Pharmacol. Bull. 31 (6), 853–859.

[B13] Donnelly S. M. Lopez N. A. Dodin I. Y. (2021). Steepest-descent algorithm for simulating plasma-wave caustics via metaplectic geometrical optics. Phys. Rev. E 104 (2), 025304. 10.1103/physreve.104.025304 34525672

[B14] Fan Z. Cai L. Wang S. Wang J. Chen B. (2021). Baicalin prevents myocardial ischemia/reperfusion injury through inhibiting ACSL4 mediated ferroptosis. Front. Pharmacol. 12, 628988. 10.3389/fphar.2021.628988 33935719 PMC8079950

[B15] Forman D. E. Butler J. Wang Y. Abraham W. T. O’Connor C. M. Gottlieb S. S. (2004). Incidence, predictors at admission, and impact of worsening renal function among patients hospitalized with heart failure. J. Am. Coll. Cardiol. 43 (1), 61–67. 10.1016/j.jacc.2003.07.031 14715185

[B16] Freda B. J. Slawsky M. Mallidi J. Braden G. L. (2011). Decongestive treatment of acute decompensated heart failure: cardiorenal implications of ultrafiltration and diuretics. Am. J. Kidney Dis. 58 (6), 1005–1017. 10.1053/j.ajkd.2011.07.023 22014726

[B17] Genheden S. Ryde U. (2015). The MM/PBSA and MM/GBSA methods to estimate ligand-binding affinities. Expert Opin. Drug Discov. 10 (5), 449–461. 10.1517/17460441.2015.1032936 25835573 PMC4487606

[B18] Jiang L. Kon N. Li T. Wang S. J. Su T. Hibshoosh H. (2015). Ferroptosis as a p53-mediated activity during tumour suppression. Nature 520 (7545), 57–62. 10.1038/nature14344 25799988 PMC4455927

[B19] Kim J. A. Wu L. Rodriguez M. Lentine K. L. Virk H. U. H. Hachem K. E. (2023). Recent developments in the evaluation and management of cardiorenal syndrome: a comprehensive review. Curr. Probl. Cardiol. 48 (3), 101509. 10.1016/j.cpcardiol.2022.101509 36402213

[B20] Klauda J. B. Venable R. M. Freites J. A. O’Connor J. W. Tobias D. J. Mondragon-Ramirez C. (2010). Update of the CHARMM all-atom additive force field for lipids: validation on six lipid types. J. Phys. Chem. B 114 (23), 7830–7843. 10.1021/jp101759q 20496934 PMC2922408

[B21] Liu H. Mei M. Zhong H. Lin S. Luo J. Huang S. (2025). Identification of anoikis-related genes in chronic kidney disease based on bioinformatics analysis combined with experimental validation. J. Inflamm. Res. 18, 973–994. 10.2147/jir.s498820 39867944 PMC11761547

[B22] Liu X. H. Chen Y. Xiao F. Dai H. Z. (2022). TSSC3 suppresses fibrosis of renal tubular epithelium by regulating PI3K/Akt signaling pathway and anoikis resistance in myofibroblasts. J. Army Med. Univ. 44 (5), 421–431. 10.16016/j.2097-0927.202107152

[B23] Ma S. Yang B. Zhao M. Li P. Fan J. Chang M. (2023). Effects of modified Huangqi Chifeng decoction on the IL-17 signaling pathway in an IgA nephropathy rat model. J. Ethnopharmacol. 307, 116220. 10.1016/j.jep.2023.116220 36750149

[B24] McCallum W. Sarnak M. J. (2023). Cardiorenal syndrome in the hospital. Clin. J. Am. Soc. Nephrol. 18 (7), 933–945. 10.2215/cjn.0000000000000064 36787124 PMC10356127

[B25] Michel J. B. (2003). Anoïkis in the cardiovascular system: known and unknown extracellular mediators. Arterioscler. Thromb. Vasc. Biol. 23 (12), 2146–2154. 10.1161/01.atv.0000099882.52647.e4 14551156

[B26] Nayar D. Agarwal M. Chakravarty C. (2011). Comparison of tetrahedral order, liquid state anomalies, and hydration behavior of mTIP3P and TIP4P water models. J. Chem. Theory Comput. 7 (10), 3354–3367. 10.1021/ct2002732 26598167

[B27] Obi Y. Kim T. Kovesdy C. P. Amin A. N. Kalantar-Zadeh K. (2016). Current and potential therapeutic strategies for hemodynamic cardiorenal syndrome. Cardiorenal Med. 6 (2), 83–98. 10.1159/000441283 26989394 PMC4790039

[B28] Özpınar G. A. Peukert W. Clark T. (2010). An improved generalized AMBER force field (GAFF) for urea. J. Mol. Model 16 (9), 1427–1440. 10.1007/s00894-010-0650-7 20162312

[B29] qiang L. G. Mu W. Jiang C. (2024). Baicalein improves renal interstitial fibrosis by inhibiting the ferroptosis *in vivo* and *in vitro* . Heliyon 10 (7), e28954. 10.1016/j.heliyon.2024.e28954 38601597 PMC11004807

[B30] Qu D. Han J. Ren H. Yang W. Zhang X. Zheng Q. (2016). Cardioprotective effects of astragalin against myocardial ischemia/reperfusion injury in isolated rat heart. Oxid. Med. Cell Longev. 2016 (1), 8194690. 10.1155/2016/8194690 26788251 PMC4695676

[B31] Rangaswami J. Bhalla V. Blair J. E. A. Chang T. I. Costa S. Lentine K. L. (2019). Cardiorenal syndrome: classification, pathophysiology, diagnosis, and treatment strategies: a scientific statement from the American heart association. Circulation 139 (16), e840–e878. 10.1161/cir.0000000000000664 30852913

[B32] Ronco C. McCullough P. Anker S. D. Anand I. Aspromonte N. Bagshaw S. M. (2010). Cardio-renal syndromes: report from the consensus conference of the acute dialysis quality initiative. Eur. Heart J. 31 (6), 703–711. 10.1093/eurheartj/ehp507 20037146 PMC2838681

[B33] Shi L. Deng Y. Luo D. Li L. Kuang X. Qi A. (2023). Exploration of the possible mechanisms of Ling Gui Zhu Gan decoction in nephrotic syndrome based on network pharmacology, molecular docking and molecular dynamics simulation. Med. Baltim. 102 (29), e34446. 10.1097/md.0000000000034446 PMC10662869 37478256

[B34] Shi M. Ning Z. (2025). *In vivo* and *in vitro* investigations of schisandrin B against angiotensin II induced ferroptosis and atrial fibrosis by regulation of the SIRT1 pathway. Sci. Rep. 15 (1), 6200. 10.1038/s41598-025-89895-0 39979353 PMC11842858

[B35] Stupack D. G. Puente X. S. Boutsaboualoy S. Storgard C. M. Cheresh D. A. (2001). Apoptosis of adherent cells by recruitment of caspase-8 to unligated integrins. J. Cell Biol. 155 (3), 459–470. 10.1083/jcb.200106070 11684710 PMC2150834

[B36] Sun S. Xun G. Zhang J. Gao Y. Ge J. Liu F. (2022a). An integrated approach for investigating pharmacodynamic material basis of Lingguizhugan Decoction in the treatment of heart failure. J. Ethnopharmacol. 295, 115366. 10.1016/j.jep.2022.115366 35551974

[B37] Sun X. Han Y. Dong C. Qu H. Yu Y. Ju J. (2022b). Daming capsule protects against myocardial infarction by promoting mitophagy via the SIRT1/AMPK signaling pathway. Biomed. Pharmacother. 151, 113162. 10.1016/j.biopha.2022.113162 35676781

[B38] Taddei M. Giannoni E. Fiaschi T. Chiarugi P. (2012). Anoikis: an emerging hallmark in health and diseases. J. Pathol. 226 (2), 380–393. 10.1002/path.3000 21953325

[B39] Van Der Spoel D. Lindahl E. Hess B. Groenhof G. Mark A. E. Berendsen H. J. C. (2005). GROMACS: fast, flexible, and free. J. Comput. Chem. 26 (16), 1701–1718. 10.1002/jcc.20291 16211538

[B40] Wang A. Yang J. Deng J. Wang K. Chen G. Lin D. (2025). Kaempferol promotes flap survival by inhibiting ferroptosis and inflammation through network pharmacology and *in vivo* experiments. Wound Repair Regen. 33 (1), e13250. 10.1111/wrr.13250 39719508

[B41] Wang F. Qin W. X. Wang Q. Deng Z. T. (2023b). Research progress of AMPK regulating ferroptosis related signaling pathway. Chin. Pharmacol. Bull. 39 (10), 1801–1805.

[B42] Wang I. C. Lin J. H. Lee W. S. Liu C. H. Lin T. Y. Yang K. T. (2023a). Baicalein and luteolin inhibit ischemia/reperfusion-induced ferroptosis in rat cardiomyocytes. Int. J. Cardiol. 375, 74–86. 10.1016/j.ijcard.2022.12.018 36513286

[B43] Wang J. Wang Y. Liu Y. Cai X. Huang X. Fu W. (2022a). Ferroptosis, a new target for treatment of renal injury and fibrosis in a 5/6 nephrectomy-induced CKD rat model. Cell Death Discov. 8 (1), 127. 10.1038/s41420-022-00931-8 35318301 PMC8941123

[B44] Wang K. Chen X. Z. Wang Y. H. Cheng X. L. Zhao Y. Zhou L. Y. (2022b). Emerging roles of ferroptosis in cardiovascular diseases. Cell Death Discov. 8 (1), 394. 10.1038/s41420-022-01183-2 36127318 PMC9488879

[B45] Wang R. Huang J. L. (2024). Mechanism of action of Linggui Zhugan Decoction in treatment of cardiovascular diseases. Chin. Traditional Herb. Drugs 55 (9), 3146–3156.

[B46] Xie Y. Song X. Sun X. Huang J. Zhong M. Lotze M. T. (2016). Identification of baicalein as a ferroptosis inhibitor by natural product library screening. Biochem. Biophys. Res. Commun. 473 (4), 775–780. 10.1016/j.bbrc.2016.03.052 27037021

[B47] Xu H. Yu S. Lin C. Dong D. Xiao J. Ye Y. (2024). Roles of flavonoids in ischemic heart disease: cardioprotective effects and mechanisms against myocardial ischemia and reperfusion injury. Phytomedicine 126, 155409. 10.1016/j.phymed.2024.155409 38342018

[B48] Yang X. Chu F. Jiao Z. Yu H. Yang W. Li Y. (2024). Ellagic acid ameliorates arsenic-induced neuronal ferroptosis and cognitive impairment via Nrf2/GPX4 signaling pathway. Ecotoxicol. Environ. Saf. 283, 116833. 10.1016/j.ecoenv.2024.116833 39128446

[B49] Yu M. Li H. Wang B. Wu Z. Wu S. Jiang G. (2023). Baicalein ameliorates polymyxin B-induced acute renal injury by inhibiting ferroptosis via regulation of SIRT1/p53 acetylation. Chem. Biol. Interact. 382, 110607. 10.1016/j.cbi.2023.110607 37354967

[B50] Zhao M. Yang B. Li L. Si Y. Chang M. Ma S. (2022). Efficacy of Modified Huangqi Chifeng decoction in alleviating renal fibrosis in rats with IgA nephropathy by inhibiting the TGF-β1/Smad3 signaling pathway through exosome regulation. J. Ethnopharmacol. 285, 114795. 10.1016/j.jep.2021.114795 34737009

[B51] Zhao M. Yin Y. Yang B. Chang M. Ma S. Shi X. (2024). Ameliorative effects of Modified Huangqi Chifeng decoction on podocyte injury via autophagy mediated by PI3K/AKT/mTOR and AMPK/mTOR pathways. J. Ethnopharmacol. 321, 117520. 10.1016/j.jep.2023.117520 38042389

